# VisRseq: R-based visual framework for analysis of sequencing data

**DOI:** 10.1186/1471-2105-16-S11-S2

**Published:** 2015-08-13

**Authors:** Hamid Younesy, Torsten Möller, Matthew C Lorincz, Mohammad M Karimi, Steven JM Jones

**Affiliations:** 1 Graphics Usability and Visualization Lab, School of Computing Science, Simon Fraser University, Burnaby, British Columbia, Canada; 2 Canada's Michael Smith Genome Sciences Centre, British Columbia Cancer Agency, Vancouver, British Columbia, Canada; 3 Research Group on Visualization and Data Analysis, Faculty of Computing Science, University of Vienna, Vienna, Austria; 4 Department of Medical Genetics, The University of British Columbia, Vancouver, British Columbia, Canada; 5 Biomedical Research Centre, The University of British Columbia, Vancouver, British Columbia, Canada; 6 Department of Molecular Biology and Biochemistry, Simon Fraser University, Burnaby, British Columbia, Canada

**Keywords:** Interactive Visualization, Sequencing Data, R-project

## Abstract

**Background:**

Several tools have been developed to enable biologists to perform initial browsing and exploration of sequencing data. However the computational tool set for further analyses often requires significant computational expertise to use and many of the biologists with the knowledge needed to interpret these data must rely on programming experts.

**Results:**

We present VisRseq, a framework for analysis of sequencing datasets that provides a computationally rich and accessible framework for integrative and interactive analyses without requiring programming expertise. We achieve this aim by providing R apps, which offer a semi-auto generated and unified graphical user interface for computational packages in R and repositories such as Bioconductor. To address the interactivity limitation inherent in R libraries, our framework includes several native apps that provide exploration and brushing operations as well as an integrated genome browser. The apps can be chained together to create more powerful analysis workflows.

**Conclusions:**

To validate the usability of VisRseq for analysis of sequencing data, we present two case studies performed by our collaborators and report their workflow and insights.

## Background

Sequencing data is the generic name for the datasets acquired using high-throughput nucleic acid sequencing techniques. This technology can be used to measure the biochemical states of cells such as the expression levels of genes or binding sites of proteins in DNA. For example, RNA sequencing (RNA-seq) measures the presence and quantity of total RNA in a cell at a given moment in time and is widely used in gene expression analysis. Another example is ChIP-sequencing (ChIP-seq) which is used to analyze protein interactions with DNA. Sequencing data come in a variety of formats. In the simplest form, each dataset represents a numerical array of the size of a genome that varies by the species (on the order of 3 billion for most mammals). Biologists are often interested in studying these data in specific regions of interest. The regions of interest are typically genomic intervals specified by features of biological interest such as the location of genes or neighbourhoods of specific genomic locations. These regions of interest are often structured in a table with rows corresponding to the regions of interest and columns being the properties of those regions, such as the genomic location, biological ID and quantified measures of enrichment of each sequencing dataset within those regions.

While computational methods to interpret these data continue to evolve, the rapidly changing computational tool set for data analysis often requires significant computational expertise to use. The Bioconductor project [1] is an open source software repository which hosts a wide range of statistical tools developed in the R programming environment [2]. Taking advantage of a rich set of statistical and graphical capabilities in R, numerous Bioconductor packages have been developed to address a variety of data analysis needs. The use of these packages, however, requires a basic understanding of the R programming/command language and an understanding of the documentation accompanying each package. As a result, R and the Bioconductor packages are primarily used by computer scientists and biologists that have a strong computational background, but remain inaccessible to most biologists who would significantly benefit from the ability to analyze such datasets. Hence, there is a clear need for a framework with an accessible user interface that allows biologists easy access to analytical tools for genomics data without requiring programming expertise.

Many useful tools have been developed in recent years for the visual analysis of biological data (e.g. MizBee [3], Pathline [4], or ChAsE [5]). What most of these tools have in common is that they have been designed to analyze and solve specific biological questions. The goal of this paper is to push the envelop toward a more general-purpose visual analysis tool that can be applied to a broad range of analyses of sequencing datasets. This is not unlike such successful attempts as VTK [6], Prefuse [7], Polaris/Tableau [8], KNIME [9], Orange [10], Lyra [11] and CSIRO Workspace [12]. These systems attempt to bring data analysis through visual means to a large audience. Most of these tools also provide some form of integration with R and enable enriching their interactive data mining and visualization components with the statistical capabilities in R, however they are mostly accessible to users who have the technical skills for R development. In addition, they do not address the specific challenges associated with sequence analysis. Simple standards, such as integration of a genome browser or support for sequencing data are missing. Many of the tools for sequence analysis are meant to be used by bioinformaticians (as opposed to for biologists), and require programming skills. Those tools aimed at the biologists, on the other hand, offer limited analytical tools and are hard to extend or generalize.

### R-based visualization systems

The lack of a graphical user interface (GUI) for the majority of the packages makes most of them inaccessible to biologists without programming expertise. Several frameworks have been developed to provide graphical user interfaces in R. Packages such as RGtk2 [13], fgui [14], R-Tcl/Tk [15], gWidget [16], JGR [17] and SciViews-R [18] allow programmers to create graphical user interfaces for command-line R packages. They have been used in general purpose packages such as Deducer [19], R Commander [20], GrapheR [21] and Rattle [22] as well as packages for biological data analysis, such as SeqGrapheR [23], limmaGUI [24], affylmGUI [25] and OLINgui [26].

With the increased popularity of web-based analysis applications, several solutions such as shiny [27], ggvis [28] and googlevis [29] have been developed to provide a web-based interface or an interactive implementation for R libraries. The graphical interfaces created by these libraries provide means to make the individual underlying R packages more accessible, however their scopes remain limited to the specific modules they are designed for and it is difficult, if not impractical, for biologists to link several modules to create more complex workflows. In addition, due to the significant coding effort required to create the graphical layout for each library and to pass the data to and from the GUI, most R libraries still remain without a graphical user interface.

### Visualization systems for biological data analysis

Several visualization systems have been developed to mitigate the dependence of biologists on programmers and allow biologists to be more involved in computational analysis tasks. Genome browsers such as UCSC [30] and IGV [31] allow users to navigate across the genome for detailed data inspection and exploration. While genome browsers are useful for viewing specific genomic regions, they are not effective for global analysis and pattern discovery. Several systems such as CisGenome [32], seqMINER [33], Cistrome [34], EpiExplorer [35], Genomic HyperBrowser [36], FlowJo [37] and SeqMonk [38] have been developed to address the need for global pattern analysis. The strength of these tools lies in their ability to connect several analysis methods in a single application, but adding newly developed analysis pipelines is not easy and researchers may find themselves waiting for state-of-the-art algorithms to be implemented within these packages. A more recent related tool is Epiviz [39] that provides an interactive genome browser and data-analysis platform for functional genomics data. A scripting interface is also provided to invoke R functions and display the results within the tool, however this extension remains accessible only to users with relevant technical skills.

### Conventional analysis workflow

The initial task in a typical analysis workflow is creating the data table for the regions of interest and sequencing data specific to the study. For each sequencing dataset, biologists compute a summary of the values of the sequencing data near each region of interest. The method for computation varies based on the type of the dataset and the study and can be as simple as adding up all values within the genomic interval, or more sophisticated methods involving machine learning (e.g. Hidden Markov Models or Baysian Networks) and non-linear normalization, but ultimately each dataset is generally summarized to one or multiple columns in a table.

Many tools (e.g. Galaxy [40] and SeqMonk [38]) have been developed to create these data tables, which come with command line or graphical user interfaces. These tools do a satisfactory job of helping biologists with the initial steps of data preparation such as quality control, sequence alignment, file format conversion and filtering. However they provide limited functionality for exploratory analyses and visualization. Thus far, such analyses can only be provided through additional programming interfaces / languages, such as R.

During the initial exploration phase, biologists frequently want to browse their datasets in a genomic context while studying the data table. Genome browsers are a popular approach for visualizing genome-scale data in which each dataset is displayed as a histogram plot or heat map, often called a "track", and multiple datasets can be viewed simultaneously by stacking these tracks. The data tables exploration is often performed in common spreadsheet applications such as Microsoft Excel and involves sorting columns and looking and verifying the information at known regions of interest. Simultaneous use of the genome browser and table view is often a tedious task requiring switching back-and-forth between various applications while copy-pasting names or locations of genomic addresses from one application to the other.

Biologists employ a variety of computational methods from simple numerical calculations on the columns to more advanced generic or domain specific statistical or machine learning algorithms. Many biologists are comfortable doing the simple calculations supported by most spreadsheet software packages. However using more advanced techniques requires familiarity with programming or scripting environments, making them inaccessible to most biologists.

Results of the computations are then illustrated in plots such as histograms, bar charts, scatter plots and heat maps. Based on those results biologists often repeat and iterate the analyses with more refined subsets, for instance with rows for which a computed p-value is lower than a certain threshold.

## Methods

In this section we will present the general framework and the design choices we made for VisRseq. We start with an overview of our design process and the tasks identified during the requirement analysis stage. We then present the R apps framework, which offers a semi-auto generated and unified graphical user interface for computational R packages and repositories such as Bioconductor [1]. We will then give an overview of the interface and its ability to chain apps together to create analysis workflows.

### Design

**Figure 1:**
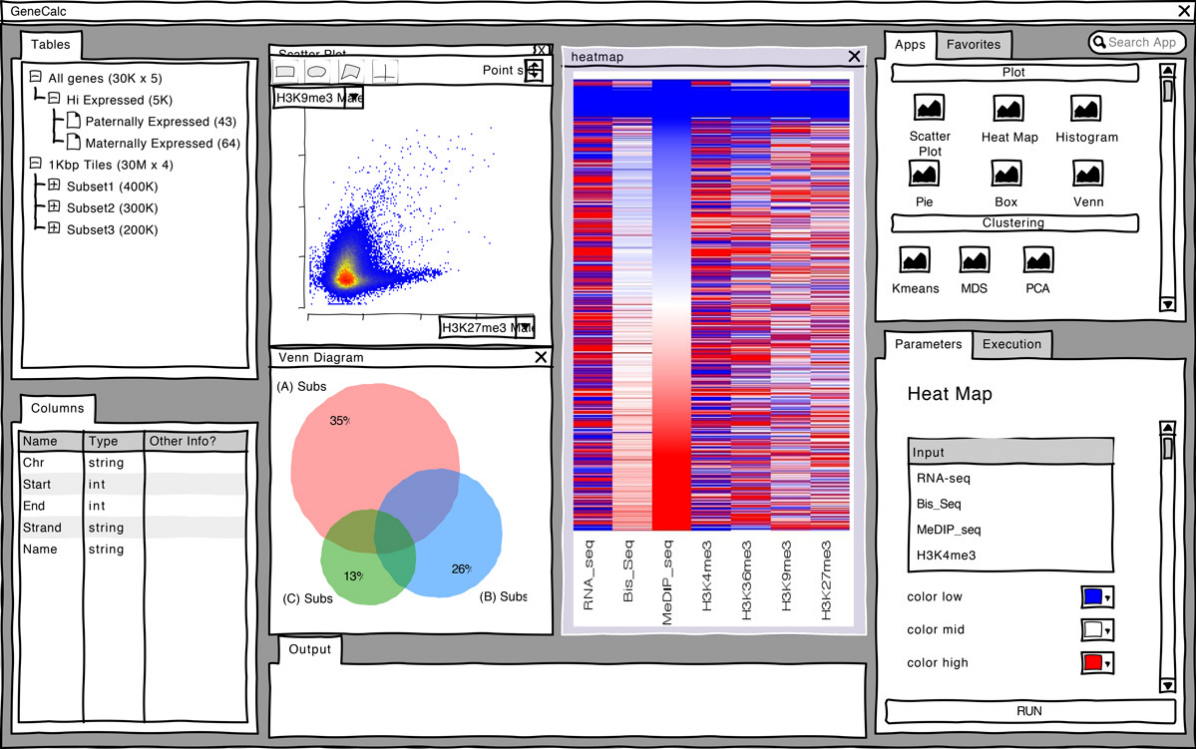
**Wire-frame prototype**. A later stage wire-frame prototype created using the WireframeSketcher software.

VisRseq was developed through an iterative user-centred design process. In our approach we followed a design study methodology [41], however as the developed solution converged toward a more general purpose framework, we realized a system's paper format would be more appropriate for presenting our results. We held formative interviews with biologists from three centres (BC Genome Sciences Centre, UBC Life Sciences Institute and later UBC Biomedical Research Centre), to understand their analysis workflow and the limitations of their existing tools. Our collaborators then evaluated the early wire-frame prototypes like the one shown in Figure 1 (created using Wireframe Sketcher [42]) and later iterated on several interactive prototypes built using Java and libraries in IGV [31] to read sequencing data formats. Our main rationale behind using a desktop platform (Java) as opposed to the a web platform (JavaScript) was being able to handle the inherently large sequencing data sets (Gigabytes) while providing an interactive user experience, a similar rationale behind popular desktop genomic viewers such as IGV [31].

As we progressed through our design we realized that all three groups of our collaborators required a more general purpose system that was capable of solving several biological data analysis problems and flexible enough to adapt to new challenges. Our requirements eventually boiled down to:

• Not requiring programming skills to use

• Inherent support for sequencing data

• Integration of a base set of analysis methods such as dimensionality reduction (PCA, MDS), clustering (Kmeans, hierarchical), and RNA-seq analysis tools.

• Integration with a genome browser

• Ease of adopting new analysis methods (extensibility)

In the following sections we will discuss our design decisions in more detail:

### R apps

There are two types of apps in VisRseq: R apps and native apps. Every R app consists of an R script file with the caller functions accompanied by a JSON file specifying the parameters to be passed to the R script. Native apps are created in Java and are pre-compiled with the framework to allow interactive graphics. They are discussed in the following section.

We had three main design goals when creating the R apps. The first was to provide an accessible interface for biologists to use libraries in R, without requiring programming expertise. The second was to allow users to link the R apps with the interactive components. The third was to minimize the effort required by R developers to create the R apps user interface for new or existing R libraries.

At the core of an R app is an R document which contains the required script to perform the desired functionality. It is up to the developer of the app to decide which parameters will be exposed to the user. These parameters will be assigned unique parameter names (exposed to the user of the app and therefore can be different from internal names). The parameter names and their types are then placed in a file in JavaScript Object Notation (JSON) format with the same name prefix and with the .json extension.

Table 1 shows the current supported variable types, the corresponding R type and the generated GUI component. An optional icon can also be specified by providing a .png file with the same name prefix.

**Table 1:** Supported types for input parameters.

variable type	R data type	GUI component
int	integer	JSpinner
double	numeric	JSpinner
boolean	boolean	JCheckBox
string	character	JTextField
string with items	character	JComboBox
filename	character	JFileDialog
color	character	JColorChooser
range-int	vector	MyRangeSlider
range-double	vector	MyRangeSlider
column	matrix	JComboBox
column-numerical	matrix	JComboBox
multi-column	data.frame	JList
multi-column-numerical	data.frame	JList
ouput-column	vector	JTextField
output-table	data.frame / matrix	JTextField

Variable type definition keywords, corresponding R data types and the generated GUI component.

Once VisRseq starts, it searches through a specific directory for all *.R files with an accompanying .json file and populates the Apps pane in the main user interface. A default gray box is used as the apps icon if an image with the app's name is not found. When the user drags an app into the workspace, the app's .json file is parsed and the graphical user interface is automatically created using Java's Swing library. In addition to providing a unified user interaction model, our intention was to minimize the effort required by developers to create apps. Unlike the previously mentioned related work on creating user interfaces for R, which required users to write the code for the actual graphical interface, we have kept the requirements to the minimum of specifying the input parameter names and types.

Once the user specifies the parameters and hits the Run button, an R session is created using the Rserve [43] library. Rserve is a TCP/IP server which allows client programs to use facilities of R from various languages including Java without the need to initialize R or link against R library. The input data table and user specified parameters are passed to the R session and the R code is executed line by line. The textual output of the R is directed to a console pane and the final graphical output is displayed in the pane assigned to the specific app. A progress animation is displayed inside the app's pane while the code is running and the user may terminate running the app by pressing the cancel button.

Apps may also have output variables. Currently we support column, table or file output. If the user specifies a name for the output (i.e. the name for the column, table or file), the output of the app is read back from the R session. A user may specify a new name to create a new column, table or file or use an existing name to overwrite one. These outputs can also be used as inputs in other apps, allowing the users to link several apps.

In addition to the auto-generated GUI, more experienced users may also browse and modify the R code by selecting the "Code" tab above the parameters pane. This will show a syntax highlighted text editor with the R code that can be edited and executed within the tool. While this is not meant to be a full featured R development environment such as RStudio [44] it is useful for more technical users as a quick way of browsing the R code and making small modifications to the apps without requiring to exit the tool.

By default the input data is loaded to the R session before the execution of the R script, but an app developer can place a line in the script with ###applyParameters to specify when exactly the parameters should be loaded. Since the R script is processed line by line, commands or structures extending over multiple lines will not execute properly. To resolve this, users can either place the lines of code inside a {{ }} block or simply put the code in a separate R file and use R's source() command to include the code.

As mentioned, our goal is to minimize the effort of R developers to create R apps. Thus the information required to create the GUI is kept to the minimum of specifying the variable's name and type (in fact specifying the type is also optional when the input is a string). However the app developer has the option to enrich the interface by specifying the following additional information:

• categories: grouping variables together. They can be collapsed or expanded by default.

• label: specifying the label shown in the GUI. If not specified, a label will be generated from the variable name by replacing the underscore "_" characters with space " " and removing the "input" prefix, if any.

• info: specifying details about the variable to be shown as a tool tip text.

• default: specifying a default value for the variable displayed in the initialized GUI.

• min / max: specifying the valid input range for the integer and numeric variables.

• items: showing a list of string items to choose from.

• ui: customizing the user interface. Currently, this is only implemented for file variables where specifying "load" or "save" will create a load or save dialog box. Additional options are planned to be added to the system to add more customization to other variable types, such as choosing between a spinner or slider for numerical columns or between combo box and radio groups for items.

### A simple R app

**Figure 2:**
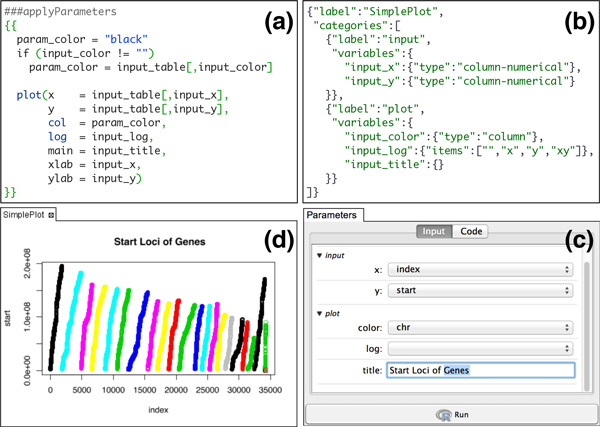
**Components of SimplePlot R app**. (a) The R code. (b) The input parameters in JSON format. (c) The auto-generated UI. (d) The graphical output after running the app.

To show the simplicity of creating R apps we walk through a simple 2D plot that uses R's default plotting functionality. Figure 2(a) shows the R code for a simple 2D plot. It takes two required parameters, input_x and input_y, the column names used for × and y, and three optional parameters, input_color for the point colors, input_log for selecting logarithmic scale and input_title for the plot title.

Figure 2(b) shows the input parameters specified in JSON format. The type specified for input_x and input_y is column_numerical which indicates the GUI should list only the numerical columns of the input table, while the type of input_color is specified as column so any column is a valid selection. For input_log a list of four strings ("", "x", "y", "xy") is specified with the first one being the default. The type for input_title is not specified so it will be considered a string input by default.

The graphical user interface generated from the parameter specification is shown in Figure 2(c) and the graphical output of running the app with example input parameters is shown Figure 2(d).

Thus far we have implemented several plotting and analysis apps as well as widely used packages from the Bio-conductor project. Among those, are DESeq [45,46] and EdgeR [47] which are popular packages used for differential expression analysis using RNA-Seq data statistics. We spent about half an hour for simple apps such as the PieChart and BarPlot apps and about two hours for the two Bioconductor apps as they required going through each package's documentation and samples. These approximate times are just for the initial creation of the apps with basic functionality and naturally we had to spend additional time iterating on each app with the users to improve the usability or to add new functionality.

### Native apps

The basic mental model of our views is a table that ties all views together. However, during our requirement analyses, our users frequently asked for an interactive interface for some of the apps to allow interactive navigation as well as brushing operators to select subsets. Since R doesn't provide such interactivity, we realized the tool would not be completely useful without interactivity at least for basic plot types. The ones with popular request were histogram, scatter plot, Venn diagram and a genome browser.

**Figure 3:**
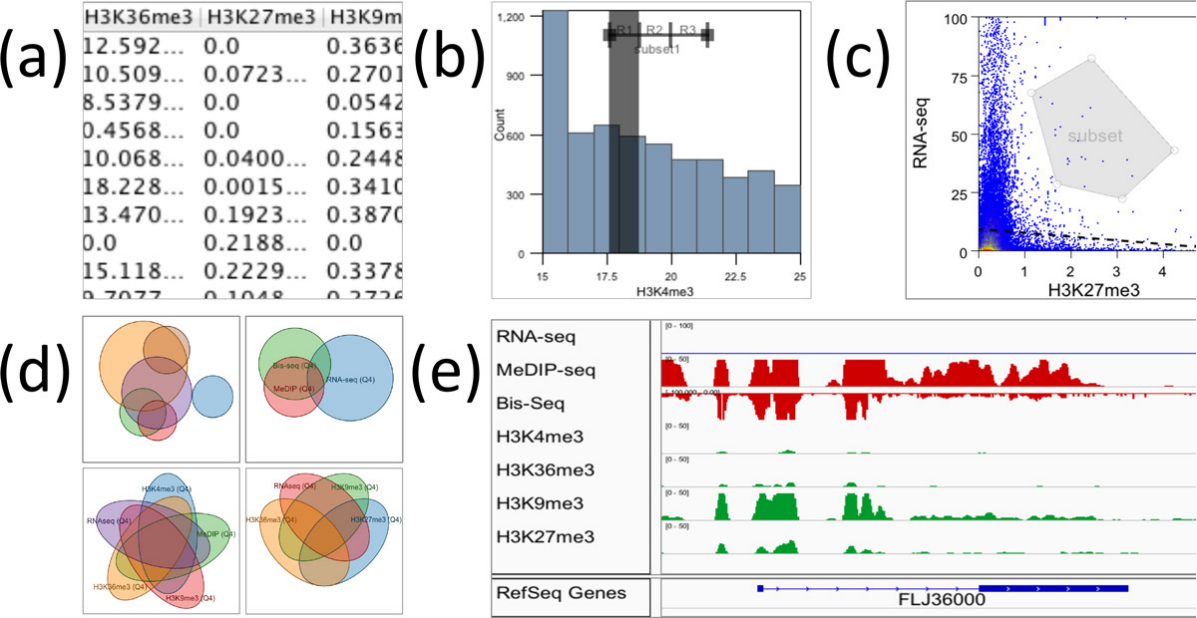
**Interactive Apps**. (a) Table view. (b) Histogram plot. (c) Scatter plot. (d) Venn/Euler diagrams. (e) IGV genome browser.

*Table view*: displays a data table in a layout common in spreadsheet software (Figure 3(a)). Clicking a column header shows a popup menu allowing users to perform several tasks such as sorting the table by that column, removing, or editing columns (e.g. changing the equation for calculated columns).

*Histogram*: provides a standard interactive frequency plot (Figure 3(b)). Any numerical table column can be used for the x-axis. The y-axis can have optional transformations such as log, cumulative distribution function and percentage. Users can perform standard panning and zooming interaction or directly specify exact values. The histogram plot offers a range filter that can be used to create a subset of the rows with their value falling within the range. Users can choose to have more than one segment for each range filter and specify whether the ranges should be equally spaced or have equal number of items. Once a filter is created it persists for that table and it is updated whenever the data values change.

*Scatter plot*: shows an interactive 2d scatter plot (Figure 3(c)). Users can specify multiple columns to the horizontal or vertical axis to effectively create a scatter plot matrix. Users can select a group of points and create a subset using the rectangle, polygon and quad filter provided. Similar to the histogram range filter, the scatter plot filters will persist and update as the data is changed.

*Venn diagram*: shows approximate area preserving Euler diagrams or symmetric Venn diagrams (up to 5 sets) for the subsets assigned to the plot (Figure 3(d)). Users can toggle between the two modes. The transition from one mode to the next is animated. The diagram is updated when any of the subsets change.

*Genome browser*: we integrated IGV [31] a widely used genome browser (Figure 3(e)). Users can load the tracks that are displayed in the genome browser into their data tables. For tables that have columns with genomic location, clicking on the rows in the Table app or on the points in the Scatter plot app navigates the Genome Browser to the corresponding genomic location.

These apps are currently implemented natively in Java. We plan to investigate the possibility of creating the interactive apps utilizing the capabilities of frameworks such as D3.js [48] and BioJS [49] to allow for easier integration of new interactive apps, however, that will require overcoming several technical challenges such as embedding JavaScript based visualizations within a Java application.

It is also worth mentioning that while we were aware of the inferiority of some of the visualization techniques (e.g. pie charts and Venn diagrams) we included them as they were requested by our collaborators and used in their workflows.

### The VisRseq graphical user interface

**Figure 4:**
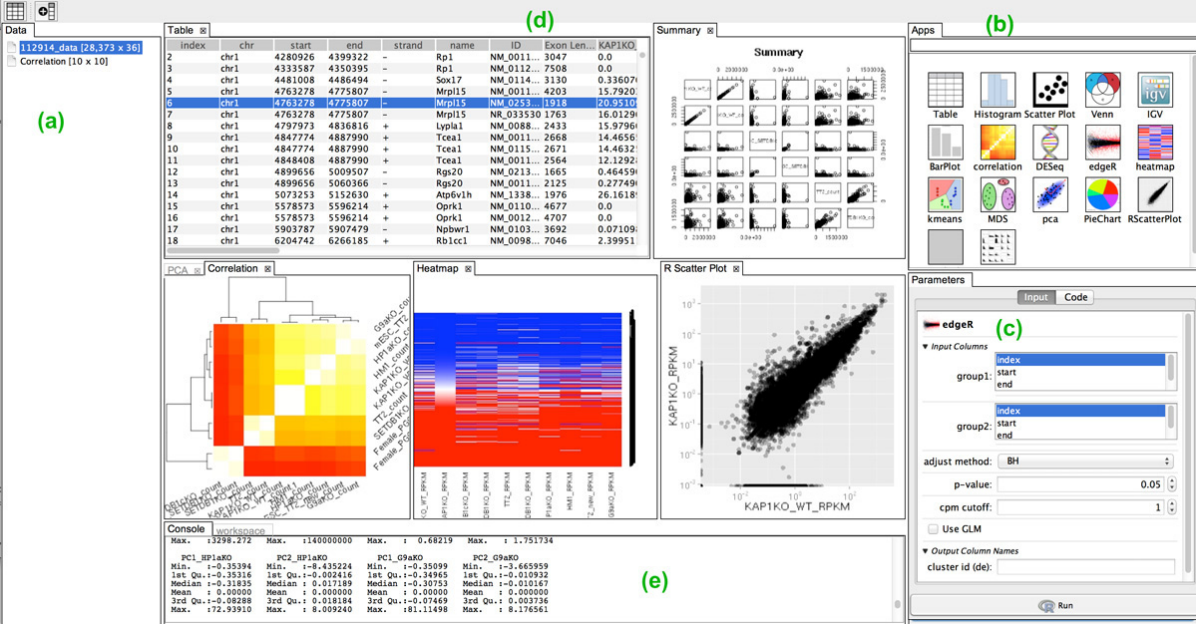
**VisRseq interface**. (a) Data pane. (b) Apps pane. (c) Parameters pane. (d) Workspace populated with several app panes. (e) Console pane.

The VisRseq graphical user interface is shown in Figure 4. It is split into several panes exposing the different functionalities provided in the framework.

At the left-hand is the Data Pane (Figure 4(a)) which depicts current data tables loaded in the system. The right-hand panel contains the Apps pane on the top (Figure 4(b)) and the Parameters pane on the bottom (Figure 4(c)). The Apps pane contains the icons for the modules available to the user; we will refer to them as "apps" throughout the rest of the paper. The Parameters pane shows the input parameters for the currently selected app. At the center is the workspace area (Figure 4(d)), where the panes for the currently running apps are laid out. Each pane is customized based on the utilities of each app, but for most apps it displays a graphical output. The textual output of apps is displayed in the Console pane at the bottom of the workspace (Figure 4(e)).

Figure 4 shows an example layout after some analysis steps. Users may change the layout of the panes to customize it based on their display size or workflow requirements. For example, we observed some users preferring to overlap the parameters and apps pane into a tabbed pane to utilize the entire horizontal space when specifying parameters. In the following sections we will describe the interface in more detail.

#### Data pane

As previously mentioned, VisRseq's internal data is a tabular data model: a collection of records with named attributes of a given data type. Users can create tables either from the feature files containing genomic regions or load text files in comma separated format. During analysis, subsets may be created through filtering, preserving the inherit hierarchy of these sets.

Table columns are either data columns created from sequencing data, calculated columns, or output columns of apps. VisRseq provides an interface with a variety of options to process and normalize sequencing data in BAM [50] and WIG [51] formats. This was one of the first features in the working prototype and was much appreciated by our collaborators as it enabled them to use their own as well as available public datasets for their analysis. Inspired by the calculation option in most spreadsheet software, we added a calculator interface using a similar syntax to Microsoft Excel that our collaborators were well familiar with. As discussed previously, R apps may also be used to create new columns or overwrite columns of an existing table (e.g. a computed cluster id or p-value).

#### Apps pane

Apps are the analysis modules of VisRseq. The Apps pane hosts an iconic view of the available apps. Individual panes for any App are created by dragging the app's icon and dropping it at the desired location in the workspace. A highlight box shows the placement of the new pane as the user drags and moves the app over the workspace area. Once an app pane is added to the workspace, the user assigns the input table to the app by dragging the desired data node from the Data pane into the app's pane.

#### Parameters pane

Whenever the user clicks on the output pane of an app, the parameters pane is updated to show the parameters for the app. As previously explained, the user interface for the parameters of R apps is automatically generated from a JSON file describing the input and output variables. We initially had the parameters within a popup dialog, but that made it hard for the user to incrementally tweak the parameters and see the results, especially for the interactive plots. We then placed the parameters side by side with each app, but realized this was an inefficient use of screen space, especially since users were usually modifying the parameters for a single app at a time.

#### Workspace pane

The panes in the workspace are laid out in a tabbed/tiled document interface similar to the layout system in rich client platforms (RCP). Our initial prototypes used a multi document interface (MDI) with fixed position for the default panes. Through user evaluations we noticed that the workflow frequently became cluttered, making it hard to organize and find the open apps. Changing to an RCP interface took time for our test users to get comfortable with, but then they expressed satisfaction with its flexible layout and how it allowed them to keep their workflow organized and clean.

#### Console pane

The console pane was required to show the textual output, progress and error messages of the apps. Since multiple R apps may be running together, we only show the textual output of the currently selected app.

### Limitations

VisRseq provides a simple way to link multiple apps, however it is currently limited to libraries that use R's standard data types as their parameters and output. More complex data types such as complex tables and custom classes cannot be integrated through the current interface options, making it difficult to link apps that require more complex data formats as parameters. One workaround is to use a file as a connecting medium but in addition to performance requirements, this approach requires writing the code to serialize the objects to and from files.

We were not able to fully automate the creation of the apps from the Bioconductor packages due to the large variety in the interfaces and input parameters for the these packages. There is no standardized meta data provided with these packages, however text mining methods might be a possibility to explore to extract those meta data from the user manuals. Still, in comparison to the previous GUIs for R, we believe we have significantly reduced the extra work by only requiring the parameter types and the function calls for each library to generate the GUI and link between different libraries in an analysis workflow.

In terms of scalability, our users have typically dealt with datasets of tens of thousands to a few million data points. Some users have worked with about 30 million data points (one data point per 100 base pair for a 3 billion base pairs genome). The framework has been robust to handle these cases, however the interactive apps become less responsive for data sets with more than few million data points. The responsiveness of R apps depends significantly based on the computational complexity of the implementation of the corresponding R packages. A simple box plot of 30 million data points takes 10-20 seconds on a typical personal computer, while a hierarchical clustering can take hours to finish.

## Validation

In our work, we differentiate between biologists and bioinformaticians. Biologists have the knowledge to analyze the sequencing data. However, they often do not have strong programming skills to use computational tools that only have a scripting interface. Bioinformaticians on the other hand have a strong algorithmic training and enough familiarity with the problem domain to develop computational tools for biologists. However, they often do not have the biological understanding required to analyze the data. The former are the target end users of this tool and the later are most suited to develop new apps.

VisRseq, as it currently stands, is used in expression analyses (i.e. RNA-seq data) and epigenomics analyses (e.g. ChIP-seq and DNA Methylation data). We believe it may also be used with most data in table format (such as microarray data), but we haven't done any evaluations on this. Also, even though our collaborators have only used the tool for mouse and human data, there is no practical limitation in using the tool for other species as long as the sequencing data is available in one of the standard formats supported by the tool (BAM [50] and WIG [51]).

To validate the usability of the tool to achieve the design goals, we conducted several case studies with collaborators who were interested in analyzing such data sets in their laboratories, two of which are presented in this section.

### Case Study 1: Gene expression in stages of mouse development

For this case study our collaborators were studying RNA-Seq data from cells from two stages of mouse development: embryonic stem cells (mESCs) and primordial germ cells (PGCs). For each development stage cells lacking expression of specific genes (knock-out/KO) as well as "control" cells with normal expression (wild-type/WT) were analyzed. Our collaborators specifically selected KO and WT datasets for four genes, "SETDB1", "KAP1", "G9a", and "HP1" to investigate the overall transcriptional correlation among cells lacking expression of these genes and the corresponding WT controls. In addition, they were also interested in identifying the genes that were up or down-regulated in knock-out cells compared to their corresponding wild-type controls. It was previously shown that these four genes play a role in the deposition of certain repressor epigenomic modifications [52-54]. Therefore, lack of expression of these genes is likely to alter the transcription of many genes. Because mouse strains are genetically diverse and each biology lab typically uses just one or a small number of the available strains to conduct their experiments, a strain-specific heterogeneity is observed among wild-type mice which complicates the comparison of KO cells derived from different mouse lines. So our collaborators were interested in characterizing this heterogeneity among those four wild-type mESC lines derived from different mouse strains.

#### Analysis 1: Identification of transcriptional correlation

**Figure 5:**
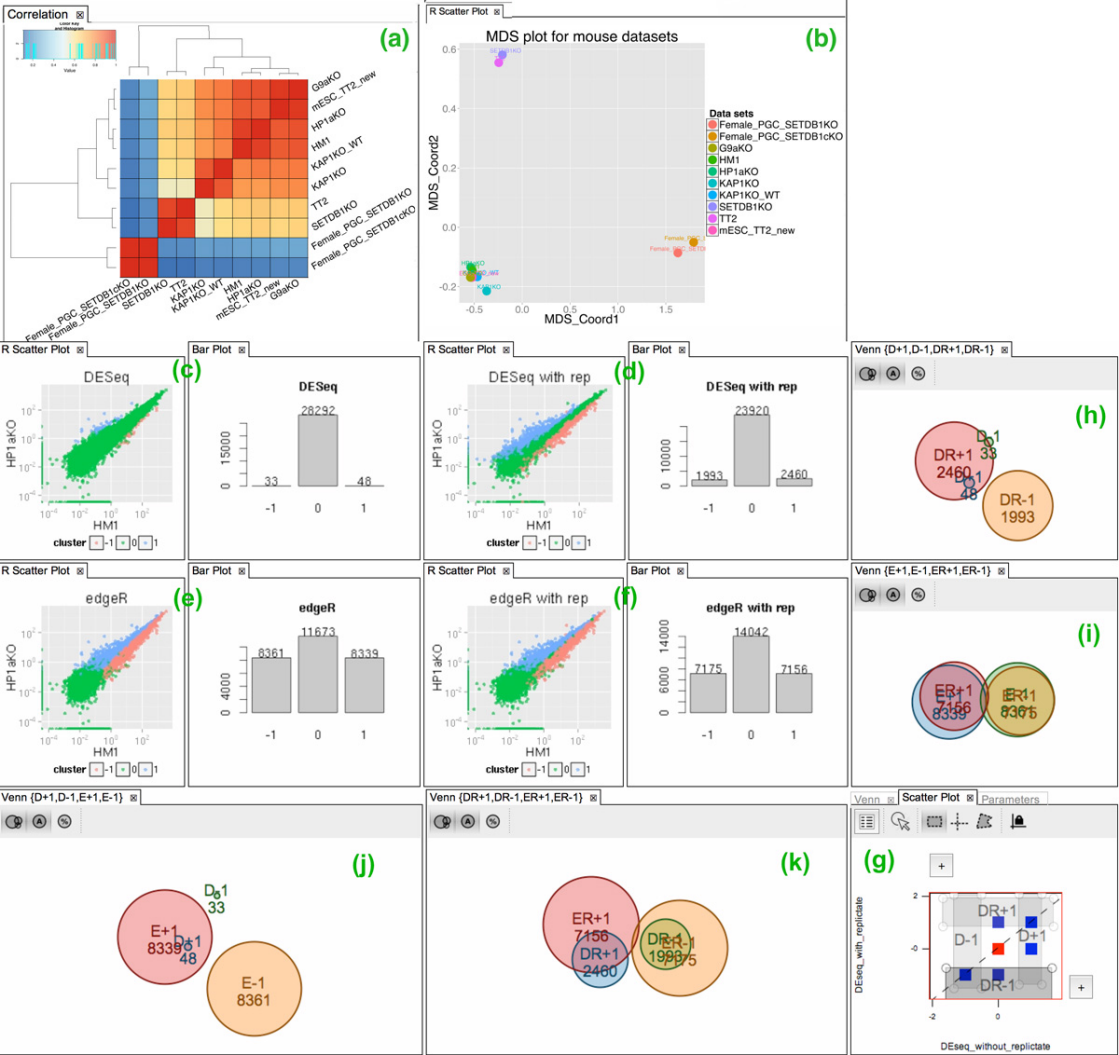
**Workflow of gene expression analysis in case study 1**. (a) Heat map with dendogram plot, and (b) MDS plot, showing the transcriptional correlation. (c-f) scatter and bar plots showing the genes up-regulated (blue color and labeled "1"), down-regulated (red color and labeled "-1") or non-differentially expressed (green color and labeled "0") based on DESeq and edgeR Apps with replicates ("DR" and "ER", respectively) or without replicates ("D" and "E", respectively). The results for pair-wise comparison of DR, ER, D, and E runs have been shown as Venn diagrams for up-regulated and down-regulated genes. (g) Native scatter plot app showing the intersection between the result of DR (DESeq with replicates) and D (DESeq without replicates) runs for up ("1") vs. down ("-1") regulated genes. The rectangle filter in native scatter plot is used to create the four subsets for Venn diagrams (h-k).

Our collaborators started by using the data import functionality to generate a data table containing the 10 RNA-seq datasets, using the RPKM (reads per kilobase per million mapped reads) statistic [55] for normalization. Subsequently, they used the "correlation" R app, and chose the Pearson coefficient option to calculate the correlation of expression profiles among these 10 columns. The result, a 10 × 10 correlation matrix, was added to the Data pane as a new data table. To visualize the correlation, they used this new table as the input of the heatmap and MDS R apps. In Figure 5(a), the heatmap output pane displays a heatmap visualization with hierarchical clustering on the correlation values illustrating the biological difference of these samples based on expression profiles of all genes. The MDS output pane added two new columns to the correlation table as the result of multi-dimensional scaling. These two columns were then used as the input of the R scatter plot app (Figure 5(b)). Both visualizations showed that the overall gene expression profile for each KO sample is more similar to its corresponding WT than any other KO sample. In addition, PGCs show significant dissimilarity of gene expression to mESCs.

#### Analysis 2: Investigation of differential expression

While the overall gene expression of KO and WT cells were fairly similar, our collaborators were interested in exploring the small fraction of genes which were up or down-regulated in KO compared to WT cells. To identify such genes, they used the R apps for two Bioconductor packages, DESeq [45] and edgeR [47], which are the state of the art methods in the genomics field for conducting differential expression analysis. Both of these methods show their best performance when biological or technical replicates exist for RNA-seq samples. Since our collaborators did not have biological replicates for any of their RNA-seq samples, they created four random sample sets for each dataset containing 30% of the data and used it as a technical replicate. They were curious to compare the results of these two methods with and without technical replicates. To do a fair comparison, our collaborators chose the Benjamini and Hochberg algorithm [56] implemented in both DESeq and edgeR to generate the false discovery rate (FDR) for each gene and applied an FDR threshold of 0.01 to identify genes showing significant changes. The output for each app was a new column indicating the predicted cluster id for each gene: "+1" for up regulated, "-1" for down regulated and "0" for no difference. The result of the two methods, DESeq and edgeR, on the data without and with replicates, are shown in Figure 5(c-f). The bar plots show the size of each predicted group and the scatter plots show the genes colored by their predicted group, on a log scale of the normalized gene expression value in the wild type (HM1) vs. knock out (HP1aKO) cells. Our collaborators then used the interactive scatter plot app to create 8 subsets of up and down regulated sets ("+" or "-") for each of the two methods ("D" or "E"), with ("R") and without replicates (Figure 5(g)). These subsets were then compared in the Venn diagrams shown in Figure 5(h-k). As shown, the edgeR result is relatively more robust in terms of the genes that are identified as up or down-regulated in HP1aKO compared to WT, whereas the DESeq result changes significantly when technical replicates were used. However, the number of genes reported by edgeR as differentially expressed genes in HP1aKO compared to WT is at least three times more than their counterparts in the DESeq analysis. This indicates that edgeR is more sensitive to the outliers, as reported previously [46]. Because the high specificity was more important than high sensitivity for our collaborators, they chose to use DESeq for their differential expression analyses because of the low false positive rate of DESeq shown by these empirical results.

### Case Study 2: Allele-specific gene expression

Each diploid cell consists of two copies of the genome, one from each parent (haplotype genome). In inbred mice, these paternal and maternal copies of the genome are identical. Hybrid mice, on the other hand, can be derived from crosses between distantly related laboratory inbred mouse strains, which differ at numerous genomic loci. Our second group of collaborators used a recently published dataset for trophoblast cells from hybrid crosses between CAST/EiJ (Cast) and C57BL/6J (B6) mice [57] and generated allele-specific (AS) profiles using the ALEA pipeline tool [58]. They were interested in a quantitative analysis of their allele-specific (AS) profiles to identify genes in RNA-seq data and/or genomic regions in ChIP-seq data showing allelic skew in one haplotype vs. the other.

#### Analysis 1: Characterization of genes with allelic imbalanced expression

**Figure 6:**
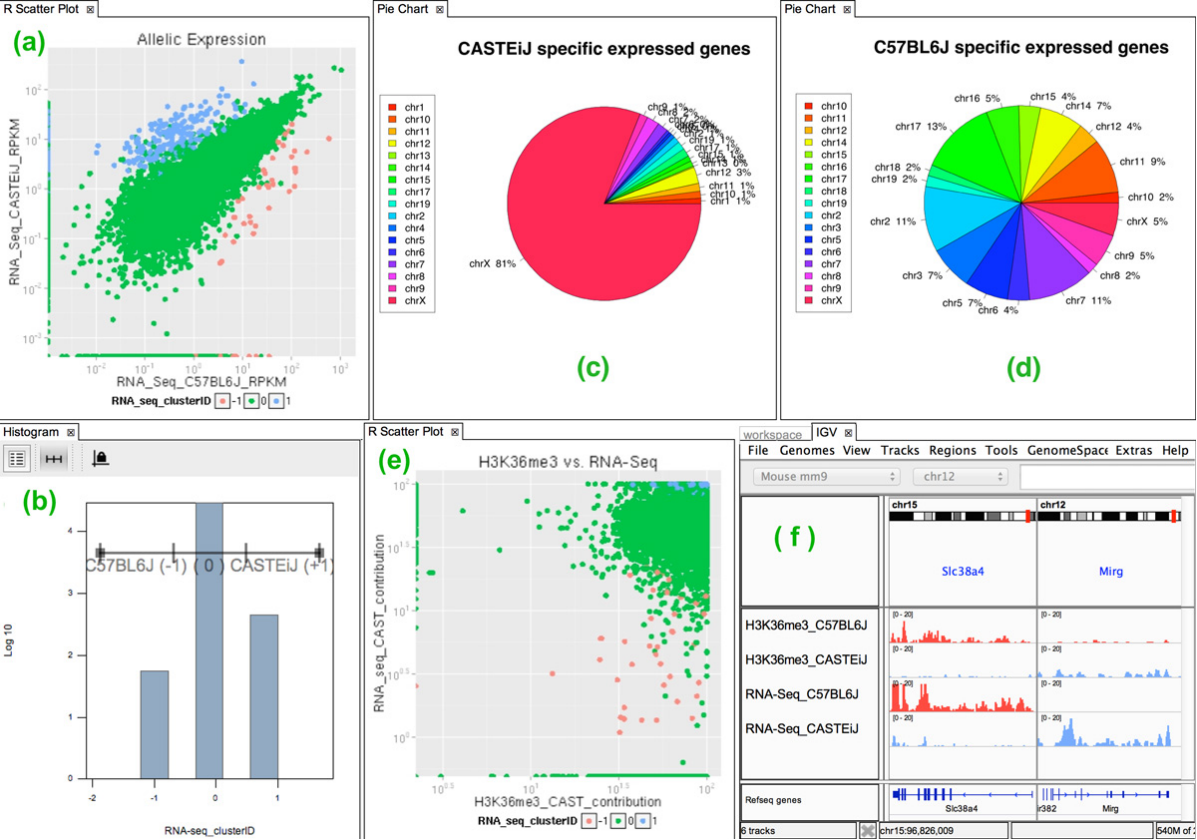
**Workflow of allele-specific gene expression analysis in case study 2**. (a) 2D scatter plot of CASTEiJ (CAST) vs. C57BL6J (B6) allelic read counts. (b) genes with allelic skew labeled by "1" for CAST and "-1" for B6 skew. (c-d) Pie charts showing chromosomal distribution of CAST vs. B6 specific expressed genes. (e) CAST contribution for H3K36me3 vs. RNA-seq. (f) genome browser view of "Slc38a4" and "Mirg" imprinted genes with mono-allelic expression in B6 and CAST.

Our collaborators started by using the DESeq R app on the AS RNA-seq data, the output of which was displayed using the R scatter plot app shown in Figure 6(a). DESeq identified 438 genes with allelic expression skew toward CAST and 55 toward B6 haplotypes. A subset of these genes were so called "imprinted genes" [59], which are known to show mono-allelic expression.

Our collaborators were curious why the number of genes that are highly expressed in CAST but not B6 was significantly higher than the number of genes that are expressed in B6 not CAST. So they opened the interactive histogram app and used the range filter to create the subsets for genes with mono-allelic expression for CAST and B6 Figure 6(b). Plotting the two subsets in separate pie charts, revealed that the majority of genes (> 350) with allelic expression skew toward CAST are located on the × chromosome (Figure 6(c)), but there is no visible pattern for B6 (Figure 6(d)). This was expected as the × chromosome is known to host a large number of maternally expressed genes (expressed in CAST haplotype here) through × chromosome inactivation [57].

#### Analysis 2: Exploration of allele-specific relation between H3K36me3 and gene expression

The epigenomic modification H3K36me3 was previously shown to be enriched in the gene body of active genes [60]. To support the above AS analysis workflow for RNA-seq data, our collaborators decided to study the potential relation between AS profiles in RNA-seq and H3K36me3 data for the genes showing allelic imbalanced expression. The CAST allelic contribution in both RNA-seq and H3K36me3 data was calculated using the tool by dividing the allelic read counts assigned to CAST to the total number of allelic reads per gene. As shown in Figure 6(e), all the 438 genes characterized in the RNA-seq analysis by DESeq as candidates with high CAST allelic contribution, show the same pattern for H3K36me3. In contrast, the genes having low CAST contribution (high B6 contribution) in RNA-seq data do not necessarily show low CAST contribution in H3K36me3 data. An IGV browser view of AS profiles for H3K36me3 and RNA-seq data (Figure 6(f)), shows two of the known imprinted genes "Slc38a4" and "Mirg", are expressed in a mono-allelic manner in B6 and CAST respectively and concurrently enriched with H3K36me3.

Taken together, this case study showed the potential of VisRseq as a visual analysis toolbox to enhance other bioinformatics tools such as ALEA by providing a visual interface to required statistical packages from R.

## Conclusions and future work

In this paper we presented VisRseq, a framework for analyzing sequencing data and creating interfaces for R libraries. By reducing the required technical expertise, VisRseq facilitates data analysis for a broader set of biologists and bioinformaticians. We created a small but diverse set of R-Apps to demonstrate VisRseq's utility and flexibility. We also provide several native apps to support interactive exploration of the data together with the output of the analysis methods. VisRseq is now being used actively by our collaborators, who report that they are able to perform their analysis pipeline more quickly and efficiently than with existing tools.

There are a number of directions for future work. We have begun to introduce VisRseq to other labs in addition to our initial collaborations. We are currently observing the use of VisRseq by these new users to evaluate and improve the usability and effectiveness for more diverse and complex analysis problems. Labs which were especially keen on working with VisRseq are employing students with bioinformatics background in order to adapt their existing R-based custom analysis modules into R apps making them more accessible to more lab members. In addition to making some of these modules available in a future release of VisRseq, this has helped us improve the process of developing new apps.

Several features are still required to support the usability of our tool. For workflows to be truly useful, there must be clear and repeatable records of what has been done, like the history system offered by tools such as Galaxy [40]. Other features include saving and loading the workspace together with the parameters, and an undo possibility. Further, reusing common parameters between apps, such as graphical variables shared among different plots, would improve the usability of the tool.

Our primary focus during the development of VisRseq was to create a tool for analyzing sequencing data. A natural extension is toward a general statistical package for other scientific data. This however requires collaboration with a broader range of scientists from different disciplines. Our vision is to eventually encourage the R package developers to also create the apps for their packages themselves and make them available through a central app repository. Although most developers would like to make their packages accessible to a broader range of users, the platform must first be adopted by a large group of biologists. Hence, for now we need to continue including a rich collection of apps to the framework ourselves.

## Competing interests

The authors declare that they have no competing interests.

## Authors' contributions

HY carried out the system design and implementation, and drafted the manuscript. TM and MMK participated in the system design. MMK and MCL participated in the coordination of case studies. TM, MMK and SJMJ supervised the project. All authors read and approved the final manuscript.
